# The Topography of Striatal Dopamine and Symptoms in Psychosis: An Integrative Positron Emission Tomography and Magnetic Resonance Imaging Study

**DOI:** 10.1016/j.bpsc.2020.04.004

**Published:** 2020-11

**Authors:** Robert A. McCutcheon, Sameer Jauhar, Fiona Pepper, Matthew M. Nour, Maria Rogdaki, Mattia Veronese, Federico E. Turkheimer, Alice Egerton, Philip McGuire, Mitul M. Mehta, Oliver D. Howes

**Affiliations:** a Department of Psychosis Studies, Institute of Psychiatry, Psychology and Neuroscience, King’s College London, London, United Kingdom; b Department of Neuroimaging, Institute of Psychiatry, Psychology and Neuroscience, King’s College London, London, United Kingdom; c Psychiatric Imaging Group, MRC London Institute of Medical Sciences, Hammersmith Hospital, Imperial College London, London, United Kingdom; d Institute of Clinical Sciences, Faculty of Medicine, Imperial College London, London, United Kingdom; e Max Planck UCL Centre for Computational Psychiatry and Ageing Research, University College London, London, United Kingdom; f Wellcome Centre for Human Neuroimaging, University College London, London, United Kingdom

**Keywords:** Functional connectivity, Negative symptoms, Positive symptoms, Resting state, Schizophrenia, Striatum

## Abstract

**Background:**

Striatal dopamine dysfunction is thought to underlie symptoms in psychosis, yet it remains unclear how a single neurotransmitter could cause the diverse presentations that are observed clinically. One hypothesis is that the consequences of aberrant dopamine signaling vary depending on where within the striatum the dysfunction occurs. Positron emission tomography allows for the quantification of dopamine function across the striatum. In the current study, we used a novel method to investigate the relationship between spatial variability in dopamine synthesis capacity and psychotic symptoms.

**Methods:**

We used a multimodal imaging approach combining ^18^F-DOPA positron emission tomography and resting-state magnetic resonance imaging in 29 patients with first-episode psychosis and 21 healthy control subjects. In each participant, resting-state functional connectivity maps were used to quantify the functional connectivity of each striatal voxel to well-established cortical networks. Network-specific striatal dopamine synthesis capacity (Ki^cer^) was then calculated for the resulting connectivity-defined parcellations.

**Results:**

The connectivity-defined parcellations generated Ki^cer^ values with equivalent reliability, and significantly greater orthogonality compared with standard anatomical parcellation methods. As a result, dopamine-symptom associations were significantly different from one another for different subdivisions, whereas no unique subdivision relationships were found when using an anatomical parcellation. In particular, dopamine function within striatal areas connected to the default mode network was strongly associated with negative symptoms (*p <* .001).

**Conclusions:**

These findings suggest that individual differences in the topography of dopamine dysfunction within the striatum contribute to shaping psychotic symptomatology. Further validation of the novel approach in future studies is necessary.

SEE COMMENTARY ON PAGE 1004

Psychotic symptoms occur across a range of mental disorders including schizophrenia, bipolar disorder, and depression. Even within a single disorder such as schizophrenia, marked symptomatic diversity exists, with clusters including positive symptoms such as hallucinations and delusions; negative symptoms such as social withdrawal and amotivation; affective symptoms; and cognitive deficits (1, 2, 3, 4, 5). Given that both symptoms and neurobiological abnormalities cross diagnostic boundaries (6), there has been an increasing focus on characterizing neuronal circuits that have transdiagnostic relevance for understanding psychopathology (7,8). Aberrant striatal dopamine signaling, and in particular, increased presynaptic dopamine synthesis capacity, has been linked to psychotic symptoms (9, 10, 11, 12, 13). Although most work has focused on the link with positive symptoms (10, 11, 12,14), it remains an open question as to whether striatal dopamine alterations are linked to other symptoms seen in psychotic disorders, such as cognitive and negative symptoms (15, 16, 17).

The striatum is a central processing hub, receiving input from almost the entire cortex (18), and plays a role in sensory, motor, cognitive, and affective processes (19, 20, 21). Thus, dysfunction in the striatum could plausibly lead to a range of heterogeneous symptoms observed in psychotic disorders. Cortical neurons largely project to discrete regions within the striatum (22), and cortical topography is mirrored striatally (18). Dopamine is a neuromodulator that plays a key role in regulating inputs and signal transmission from the striatum (23). Given this preserved topographical mapping of cortical inputs, the precise location of dopamine dysfunction within the striatum is likely to determine which particular corticostriatal circuits are affected (9) and, in turn, may be expected to shape symptomatology.

Improvements in the resolution of positron emission tomography (PET) scanners have meant that greater spatial precision is possible when imaging striatal dopamine. This has led to the finding that dopamine dysfunction in schizophrenia is not uniform across the striatum, but rather shows significant spatial variability (9,24, 25, 26). When investigating dopamine function with typical anatomically based parcellation methods, however, a high degree of correlation is observed between striatal subdivisions (26). This collinearity precludes investigation of the hypothesis that spatial variability may shape symptomatology (26).

Resting-state functional magnetic resonance imaging (rs-fMRI) can be used to quantify the functional connectivity between two brain regions or voxels by quantifying the correlation between the neural activity time series within each region (27,28). This allows one to map individualized corticostriatal functional connectivity and generate connectivity-defined striatal parcellations. Striatal parcellations derived from participant-specific corticostriatal connectivity patterns may better capture the functional topography of the striatum compared with standard group-level anatomically based striatal parcellations. When combined with PET imaging, this method may lead to greater orthogonality between dopamine measures within striatal subdivisions compared with anatomically based methods, and thereby allow testing of the hypothesis that spatial variation in dopamine function across the striatum influences symptomatology.

In the present study, we used rs-fMRI to map functional corticostriatal connections in patients presenting with first-episode psychosis and PET to examine striatal dopamine synthesis capacity within the same individuals. By combining rs-fMRI and PET, we were able to evaluate dopamine function within subdivisions of the striatum that had been defined on the basis of their cortical connectivity at an individual level. We first validated this method by comparing with typical anatomically based methods of parcellating the striatum in terms of test-retest reliability and subdivision orthogonality (29,30). We next used this method to examine whether dopamine synthesis capacity within individualized connectivity-defined regions correlated with Marder factor scores (factor analysis–derived subscales of the Positive and Negative Syndrome Scale) (3) and investigated whether relationships between dopamine-symptom associations are significantly different between subdivisions.

## Methods and Materials

### Overview

**Figure 1:**
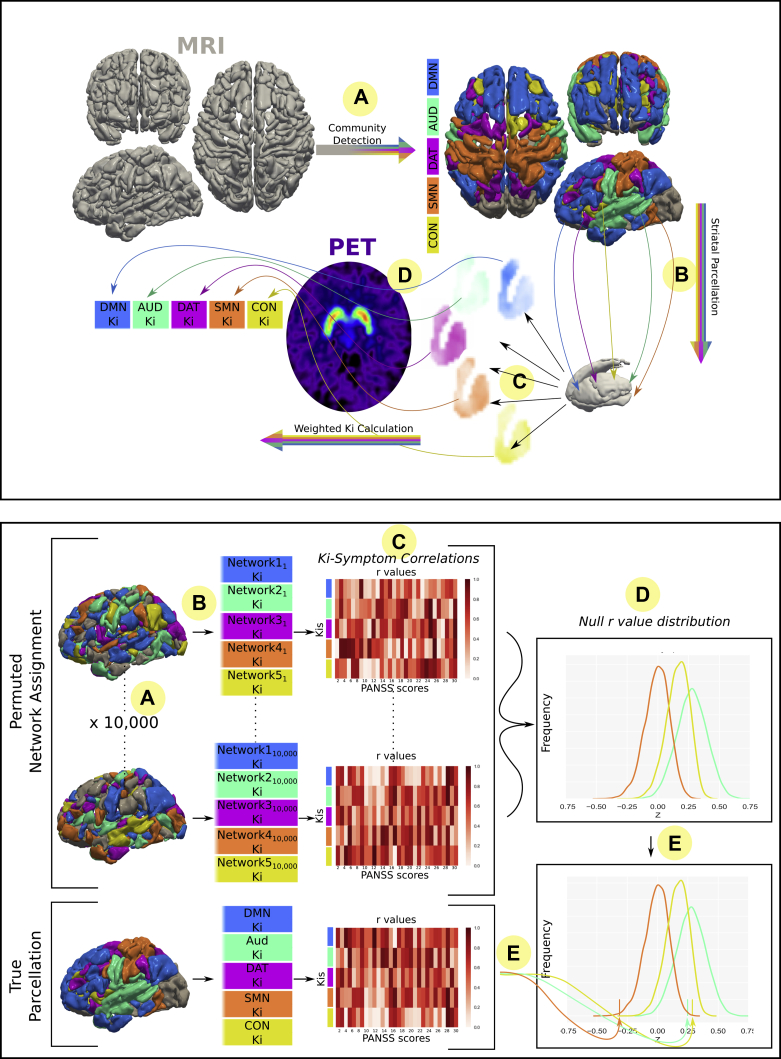
Overview of methods. Top: Participants receive resting-state magnetic resonance imaging (MRI) and ^18^F-DOPA positron emission tomography (PET) scans. **(A)** Cortical nodes are assigned to networks based on corticocortical resting-state functional connectivity. **(B)** Connectivity of each striatal voxel to these cortical networks is calculated. **(C)** Weighted striatal connectivity maps are produced for each network (see Figure S2). **(D)** Voxelwise dopamine synthesis capacity (Ki^cer^) (Ki) maps weighted by these striatal connectivity maps to give a Ki^cer^ value for each network. Bottom: Significance testing of Ki^cer^-symptom relationships using a permutation testing approach. In addition to permuting at the level of participants (not pictured), cortical regions of interest were permuted to generate null distributions: **(A)** Cortical regions of interest shuffled into random networks 10,000 times. **(B)** These shuffled network sets were used to calculate Ki^cer^ with the same method described above in the top panel. **(C)** Symptom-Ki^cer^ correlations were calculated for each null set of Ki^cer^. **(D)** Null distribution was created from repeating step **(C)** for each of the null network sets. **(E)** True symptom-Ki^cer^ correlation values were compared with null distribution to test statistical significance. AUD, auditory network; CON, cingulo-opercular network; DAT, dorsal attention network; DMN, default mode network; PANSS, Positive and Negative Syndrome Scale; SMN, sensorimotor network.

A total of 29 first-episode psychosis patients and 21 healthy control subjects received an ^18^F-DOPA (3,4-dihydroxy-6-[18F]fluoro-L-phenylalanine) PET scan and an MRI scan. Clinical ratings were performed using the Positive and Negative Syndrome Scale by a consultant psychiatrist blinded to imaging outcome measures. For each participant, we used functional connectivity between cortical resting-state networks and the striatum to generate individualized connectivity-defined striatal parcellations. We then combined this with PET data to calculate the dopamine synthesis capacity (Ki^cer^) for these connectivity-defined parcels. We next combined test-retest datasets for both ^18^F-DOPA and resting-state MRI (31,32) to compare the reliability and collinearity of Ki^cer^ values calculated using this method with those calculated using a traditional anatomically based parcellation. We then investigated whether dopamine function within these connectivity-defined striatal subdivisions showed a relationship with symptomatology in patients, and evaluated the specificity and statistical significance of any observed relationships using a permutation testing approach. The analysis approach is summarized in Figure 1 and described in detail in the Supplement.

### Participants

Participants were experiencing a first episode of psychotic illness, meeting ICD-10 criteria (33), and were antipsychotic naïve (*n =* 11), antipsychotic free for at least 6 weeks (*n =* 16), or minimally treated for <2 weeks (*n =* 2). Age-matched (within 5 years) healthy control subjects were recruited from the same geographical area through local media advertisements. Control subjects had no previous or current history of psychiatric illness (assessed by the Structured Clinical Interview for DSM-IV Axis I Disorders), no concurrent psychotropic medication use, and no family history of psychosis. See the Supplemental Methods and previously published reports for further details regarding recruitment and assessment (34,35). Some of the data for these participants have been previously reported (14,34, 35, 36).

### Image Acquisition

Participants received an ^18^F-DOPA PET scan, providing a measure of striatal dopamine synthesis capacity (37). The cerebellum was used as a reference region, and voxelwise parametric images of Ki^cer^ were constructed from movement-corrected images using a wavelet-based Patlak approach (see Figure S1) (38). We also determined Ki^cer^ for limbic, associative (the pre- and postcommissural caudate, and precommissural putamen), and sensorimotor (postcommissural putamen) striatal subdivisions, using the anatomically defined approach outlined by Martinez *et al.* (30). Participants also received an 8.5-minute rs-fMRI scan on a 3T GE Signa MR scanner (GE Healthcare, Milwaukee, WI). See the Supplemental Methods for further details.

### Image Analysis

#### Cortical Network Definition

fMRI signal time series were extracted from the 333 cortical regions (nodes) of the Gordon cortical atlas (a network parcellation based on fMRI functional connectivity patterns observed in a sample of 120 healthy young adults). Functional connectivity between every pair of nodes was defined as the pairwise *z*-transformed Pearson correlation coefficients between the fMRI time series of each region (39) and was used to define a 333 × 333 functional connectivity matrix for each participant. The Louvain community detection algorithm was then employed on this whole-cortex connectivity matrix, to group each cortical node into nonoverlapping communities in a manner that maximizes the modularity of the final network (40). The detected communities corresponded to well-recognized resting-state networks: the default mode network (DMN), sensorimotor network, cingulo-opercular network (CON), dorsal attention network (DAT), auditory network (AUD), and visual network (Figure S2). The visual network was excluded from subsequent analyses given its relative lack of direct connections with the striatum (41). Analysis was performed with in-house Python code (Python Software Foundation; Python Language Reference, version 3.7; http://www.python.org).

#### Striatal Parcellation and PET Integration

An individualized probabilistic approach was employed. For each participant, for each cortical network identified above, each striatal voxel was assigned a connectivity score between 0 and 1 based on its mean connectivity to all nodes within that network (see Figure S3). A weighted striatal map was thereby constructed for each of the networks identified. We used a probabilistic (as opposed to a winner-takes-all) approach, given the fact that although corticostriatal pathways run in parallel, there is a high degree of overlap (18). These striatal maps were then overlaid on the PET voxelwise Ki^cer^ maps to enable the calculation of network-specific Ki^cer^ values.

#### Reliability and Orthogonality

A total of 16 (8 participants) test-retest PET maps were available from a previous study (31). These were paired with 80 (40 participants) test-retest resting-state scans from the Human Connectome Project (32). Using the methods described above, individual connectivity-defined Ki^cer^s were calculated for each PET–resting-state pair. This provided five test-retest datasets in which each set contains the same 8 PET participants but different, nonoverlapping Human Connectome Project participants. For each PET participant, Ki^cer^ values were also calculated using the widely used anatomically defined Martinez *et al.* (30) striatal parcellation. Intraclass correlation coefficients (ICCs) between test and retest scans were calculated using the R package irr 0.84.1 (42). We employed the same method used in the original study of ^18^F-DOPA test-retest reliability using the method of Shrout and Fleiss (43) with 2-way random subject effects, fixed session effects, and parcel Ki^cer^s considered the average of individual voxels (31). The ideal method for assessing reliability would involve PET and MRI scans obtained from the same individuals, as the fact that the PET and MRI scans are from different individuals has the potential to reduce the reliability of the method. As such, this analysis provides a lower bound on the method used for calculating subdivision specific Ki^cer^ values. We also performed an analysis of solely the MRI data for the same 80 Human Connectome Project scans, in which we investigated test-retest reliability of mean connectivity strength for each striatal subdivision (i.e., the reliability of the weighting that is subsequently used to calculate the Ki^cer^ values).

Using data from the current study, we investigated whether the connectivity-defined subdivisions showed greater orthogonality in terms of Ki^cer^ values compared with anatomically defined subdivision Ki^cer^s. Specifically, the correlation coefficients between subdivisions across all participants defined with one method were compared with the correlation coefficients between subdivisions defined using the other method, using the method of Silver *et al.* (44) implemented in the R package *cocor* (version 1.1-3).

#### Dopamine-Symptom Relationships

Based on previous findings (13,34,45), we tested the hypothesis that Ki^cer^ would be linearly related to severity of symptoms. Symptoms were grouped according to the Marder five-factor model (3), and Pearson correlation coefficients were calculated between each factor and each network-specific Ki^cer^.

Statistical significance was assessed using two separate permutation testing approaches—participant level permutations and cortical node permutations. A permutation testing approach was employed, as this is nonparametric and makes minimal assumptions regarding the structure of the data. In both approaches, the relationship between all five Marder factors and the five connectivity parcellation–defined Ki^cer^s were tested, and false discovery rate (FDR) correction for the 25 tests undertaken was performed (46). The first approach involved permuting at the participant level (i.e., shuffling the mapping between participant-specific symptom scores and Ki^cer^ values), generating a null distribution by calculating correlation coefficients after permuting Ki^cer^ values while keeping symptom scores fixed (10,000 permutations). We then tested statistical significance by comparing the correlation coefficients between subdivision Ki^cer^ and symptom scores observed in the actual data, with the coefficients observed in the permuted data.

The participant level approach, however, does not account for the general relationship between whole-striatum Ki^cer^ and total symptoms, in that any significant findings could reflect a general association between whole striatal Ki^cer^ and symptoms in general, rather than a relationship between a symptom domain and a Ki^cer^ from a specific connectivity-defined parcellation. Therefore, we also employed a separate approach in which we permuted the cortical nodes assigned to networks (10,000 permutations), thereby creating a null distribution that retained the relationship with mean striatal dopamine synthesis capacity (Figure 1B). With this approach, we were able to test whether an observed subdivision Ki^cer^-symptom correlation was truly specific to that identified subdivision over and above the general striatal Ki^cer^-symptom relationships present in the data. The use of both permutation approaches therefore represents a particularly robust analysis of the statistical significance of our observed dopamine-symptoms correlations; accounting for outliers, skewed data distributions, and allowing for testing of the specificity of the subdivision-symptom relationships.

An aim of the current study was to determine whether the connectivity-based approach had the ability to highlight dopamine-symptom relationships that were distinct between subdivisions. We therefore tested whether symptom-dopamine associations were significantly different between subdivisions. For a given symptom domain, for each possible pair of subdivisions, we calculated the true absolute difference between subdivision-symptom correlation coefficients. We then determined statistical significance by comparing this true difference with the equivalent differences observed in the (participant level) permuted data.

#### Patient–Control Subject Differences in Connectivity and Dopamine Function

We also tested for differences in striatocortical connectivity between patients and control subjects, as this could potentially lead to differences in the connectivity-based parcellations. We first investigated whether differences existed in subdivision weightings. For each individual, for each connectivity-defined subdivision, we calculated the mean of all the connectivity values within that subdivision. We then compared these values between patients and control subjects using an independent samples *t* test.

In addition to differences in overall weightings, there also exists the possibility that patients and control subjects may differ in terms of the spatial layout of the connectivity-based subdivisions. In order to investigate this, for each subdivision we tested whether there was a difference in weightings for patients compared with control subjects in any of the three axes (x, y, z). For the x-axis, for example, we multiplied each subject’s 3-dimensional connectivity-based subdivision by a 3-dimensional matrix that showed a linear progression in value only across the x-axis; we then summed the values for that individual’s newly weighted subdivision and used this value in an independent samples *t* test between patients and control subjects, allowing us to see whether patients or control subjects showed a tendency to show greater laterality along the x-axis in terms of this subdivision’s weighting (see Figure S4).

We also examined patient–control subject differences in Ki^cer^ for each striatal subdivision using an Independent samples *t* test. Nineteen of the patients and 12 of the control subjects were included in a previously reported study (34), in which raised dopamine synthesis capacity was present only in responders to antipsychotic treatment; therefore, we also investigated patient and control subject Ki^cer^ differences after only including patients subsequently determined to be antipsychotic responders (*n =* 11).

### Data Availability

Code used for analysis is freely available (https://github.com/robmcc10/dopamine_symptoms_bp_cnni). Data are available from the authors upon request.

## Results

### Participants

A total of 50 participants took part in the study (21 control subjects and 29 patients). Demographic details are given in Table 1. Mean total Positive and Negative Syndrome Scale score for patients was 66.7 ± 20.7.

**Table 1:** Demographic Details of Study Participants

Variable	Control Subjects (*n* = 21)	Patients (*n* = 29)	*p* Value
Male	13 (62)	22 (76)	.45^a^
Age, Years	23.3 ± 3.4	25.5 ± 4.2	.06^b^
Ethnicity, White British	14 (67)	10 (35)	.05^a^
Current Smoker	5 (24)	12 (41)	.32^a^
Right-handed	19 (90)	26 (90)	.99^a^
Education, Years	16.8 ± 1.9	14.2 ± 3.4	.002^b^
Medication Status			
Antipsychotic naïve	NA	11 (38)	NA
Minimally treated^c^	NA	2 (7)	NA
Antipsychotic free	NA	16 (5)	NA
Diagnosis			
Schizophrenia	NA	15 (52)	NA
Bipolar	NA	12 (41)	NA
Other	NA	2 (7)	NA
Days Between PET and MRI Scans	70 (24–250; 4–733)	8 (3–24; 1–371)	<.001^d^
PANSS Total	NA	66.7 ± 20.7	NA
PANSS Positive	NA	17.6 ± 6.9	NA
PANSS Negative	NA	15.1 ± 6.3	NA
PANSS General	NA	34.0 ± 10.1	NA
Injected Activity, MBq	152.9± 12.6	143.5 ± 7.4	.01^b^

Data are expressed as *n* (%), mean ± SD, or median (interquartile range; range).

MRI, magnetic resonance imaging; NA, not applicable; PANSS, Positive and Negative Syndrome Scale; PET, positron emission tomography.

a χ^2^ test.

b Independent samples *t* test.

c Receiving antipsychotic medication for 2 weeks or less.

d Kruskal-Wallis test.

### Cortical Network Assignment and Striatal Connectivity Maps

**Figure 2:**
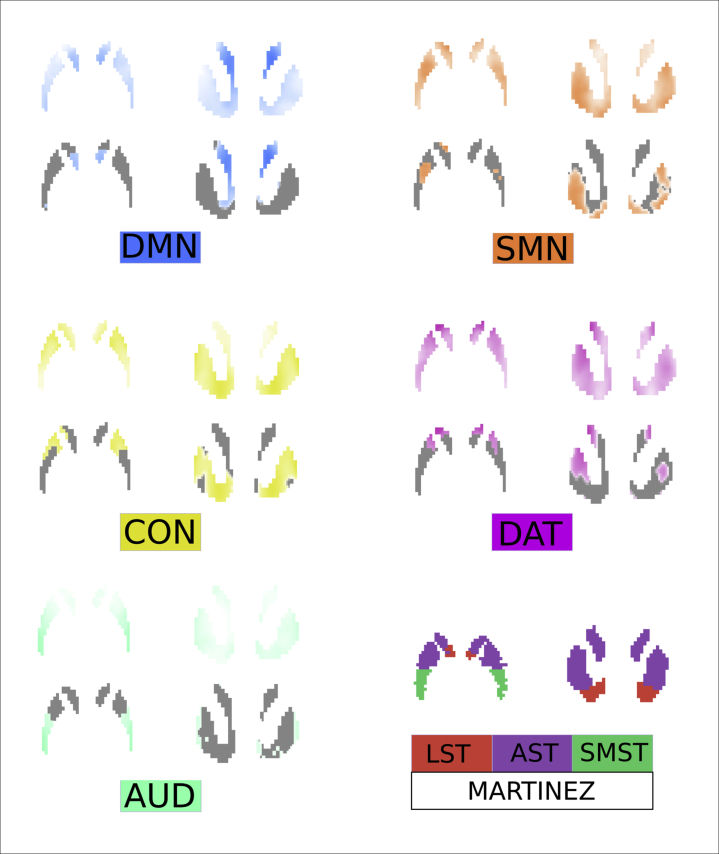
Connectivity-defined striatal maps. Striatal connectivity maps were used to weight voxelwise Ki^cer^ maps and generate network-specific Ki^cer^s. Group-averaged maps are shown, whereas individualized maps were used in practice. Maps are normalized by total connectivity strength. Greater intensity of color indicates that a voxel displays greater connectivity to the cortical network in question. In order to show differences between networks more clearly, thresholded maps are also shown (retaining only top 35% of voxels). The anatomically defined Martinez *et al.* (30) parcellation is also shown at the bottom right. AST, associative striatum; AUD, auditory network; CON, cingulo-opercular network; DAT, dorsal attention network; DMN, default mode network; LST, limbic striatum; SMN, sensorimotor network; SMST, sensorimotor striatum.

The community detection algorithm assigned nodes to 5 separate networks, and these corresponded to well-recognized resting-state networks (DMN, AUD, DAT, sensorimotor network, CON) (see Figure S2). The connectivity between these networks and the striatum was calculated at the individual-participant level, although for display purposes, group-averaged maps are shown in Figure 2.

### Reliability and Comparison With Existing Parcellation Methods

When examining reliability and orthogonality, we compared our individualized connectivity-based approach to an anatomical approach (29,30). Reliability using both methods was good, but the ICC was generally higher using the connectivity-based approach, in which it ranged from 0.73 to 0.78, compared with 0.65 to 0.80 for the anatomically defined subdivisions (see Supplemental Results). ICCs of solely the rs-fMRI–based connectivity parcellations were fair to good for all subdivisions (ICCs = 0.48–0.65) except the DAT (ICC = 0.32) (see Supplemental Results) (47).

**Figure 3:**
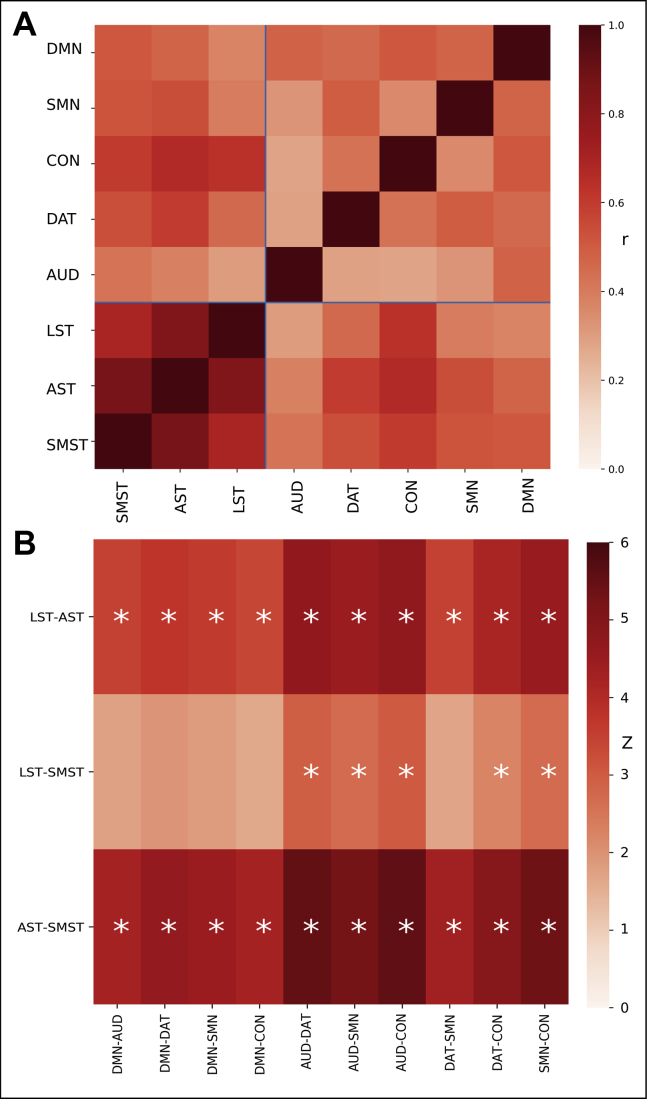
Comparison of connectivity-defined and anatomically defined subdivisions. **(A)** Heatmap displaying correlation coefficients between Ki^cer^ values for different subdivisions. There is greater orthogonality between connectivity-defined subdivisions (auditory network [AUD], dorsal attention network [DAT], cingulo-opercular network [CON], sensorimotor network [SMN], default mode network [DMN]) (*r*_p_ = .23–.67) compared with anatomically defined subdivisions (limbic striatum [LST], associative striatum [AST], sensorimotor striatum [SMST]) (*r*_p_ = .71–.91). **(B)** Comparing the magnitude of these intramethod correlation coefficients, these are significantly lower (∗*p <* .05) for the connectivity-based method (i.e., indicating greater orthogonality) for all but 5 of the 30 comparisons.

In addition to reliability, orthogonality between subdivision Ki^cer^s is required for the investigation of unique subdivision-symptom relationships, as a high degree of correlation between subdivisions effectively precludes the identification of relationships specific to a subdivision. Correlations between subdivision Ki^cer^s demonstrated that the anatomical subdivisions showed highly collinear relationships (Pearson's correlation coefficient [*r*_p_] = .76–.92), while in contrast, the connectivity-defined subdivisions showed much greater orthogonality (*r*_p_ = .23–.65). The connectivity-based approach showed numerically greater orthogonality for all 30 possible comparisons between the methods, which was statistically significant for 25 of these (Figure 3).

### Symptom-Dopamine Relationships

**Figure 4:**
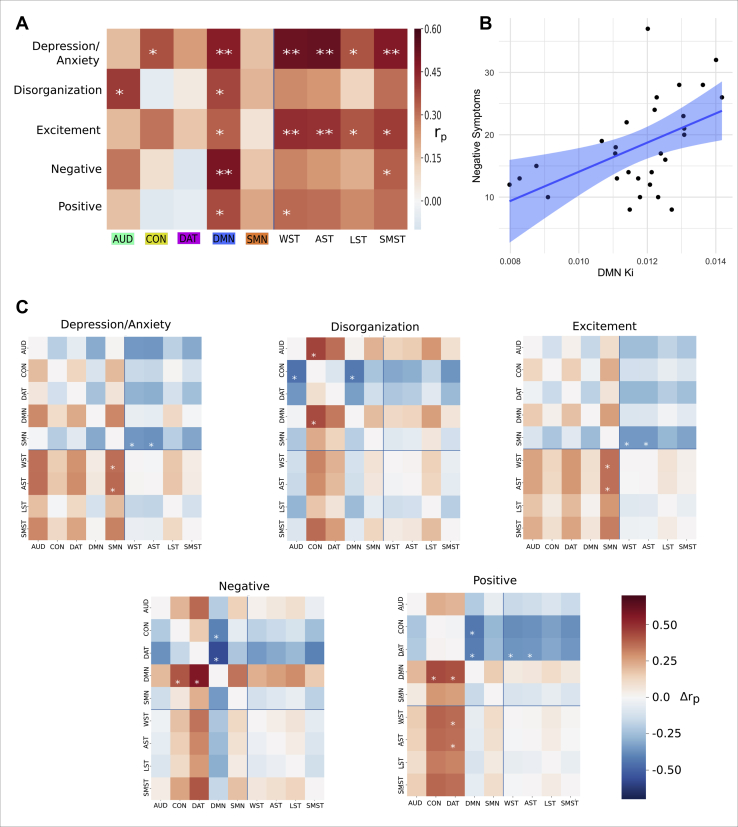
Relationships between dopamine synthesis capacity (Ki^cer^) and psychotic symptoms. **(A)** Associations between Positive and Negative Syndrome Scale Marder factors and striatal Ki^cer^ (Ki) across different striatal subdivision regions. Heatmap displays *r*_p_ values. Statistical significance calculated by permuting participants. ∗*p* < .05, ∗∗*p* < .05 (false discovery rate corrected). **(B)** The strongest association observed using the connectivity-based approach was between default mode network (DMN)–Ki^cer^ and the Marder negative factor score (*r*_p_ = .49, *p* = .009). **(C)** Heatmaps illustrating the extent to which symptom-Ki^cer^ associations differ between subdivisions. Positive values indicate that the row subdivision shows a greater association with the Marder factor than the column subdivision. ∗*p* < .05. Connectivity-defined subdivisions: auditory network (AUD), cingulo-opercular network (CON), dorsal attention network (DAT), DMN, and sensorimotor network (SMN). Anatomically defined subdivisions: whole striatum (WST), associative striatum (WST), limbic striatum (LST), and sensorimotor striatum (SMST).

The associations between Marder factor scores and subdivision Ki^cer^s are displayed in Figure 4. When permuting participants (Figure 4A), significant positive associations were observed between the AUD Ki^cer^ and the disorganization factor (*r* = .40, *p =* .01, FDR-corrected *p =* .11), and between the CON Ki^cer^ and the depression/anxiety factor (*r*_p_ = .37, *p =* .028, FDR-corrected *p =* .11). The DMN Ki^cer^ showed a significant association with all factors (depression/anxiety: *r*_p_ = .47, *p =* .003, FDR-corrected *p =* .04; disorganization: *r*_p_ = .38, *p =* .02, FDR-corrected *p =* .11; excitement: *r*_p_ = .33, *p =* .03, FDR-corrected *p =* .11; negative: *r*_p_ = .49, *p =* .0009, FDR-corrected *p =* .02; positive: *r*_p_ = .37, *p =* .025, FDR-corrected *p =* .11) (see Figure 4B).

In terms of the anatomically defined regions, the whole striatum showed significant associations with depression/anxiety (*r*_p_ = .53, *p =* .002, FDR-corrected *p =* .02), excitement (*r*_p_ = .43, *p =* .01, FDR-corrected *p =* .048), and positive (*r*_p_ = .32, *p =* .048, FDR-corrected *p =* .09) factors. The three subdivisions were all significantly associated with depression/anxiety and excitement factors. The associative subdivision showed the strongest relationship (depression/anxiety: *r*_p_ = .53, *p =* .002, FDR-corrected *p =* .02; excitement: *r*_p_ = .42, *p =* .01, FDR-corrected *p =* .048), followed by the sensorimotor subdivision (depression/anxiety: *r*_p_ = .48, *p =* .006, FDR-corrected *p =* .04; excitement: *r*_p_ = .40, *p =* .02, FDR-corrected *p =* .07), and the limbic subdivision (depression/anxiety: *r*_p_ = .35, *p =* .03, FDR-corrected *p =* .08; excitement: *r*_p_ = .35, *p =* .03, FDR-corrected *p =* .08). Symptom-subdivision relationships remained statistically significant in a sensitivity analysis excluding the two minimally treated participants (Figure S5), and also when visual network nodes were included (Figure S6).

As discussed above, permuting participants does not account for a more general overall striatal Ki^cer^-symptom association. When using the cortical node permutation approach, the DMN Ki^cer^ still showed significant associations, those being with the negative (*p =* .0015, FDR-corrected *p =* .038), depression/anxiety (*p =* .023, FDR-corrected *p* = .19), and disorganization (*p =* .034, FDR-corrected *p =* .21) factors. The AUD Ki^cer^ also showed an association with the disorganization factor (*p =* .022, FDR-corrected *p =* .19) (Figure S7).

As shown above, the connectivity-based approach led to significantly greater orthogonality between subdivisions compared with the anatomical parcellation. We examined whether this was accompanied by symptom-subdivision relationships that were significantly different from one another (Figure 4C). For the depression/anxiety and excitement Marder factors, the connectivity-defined sensorimotor network subdivision showed a significantly lower correlation coefficient compared with the whole striatum (depression/anxiety: *p =* .04; excitement: *p =* .04) and associative striatum (depression/anxiety: *p =* .03; excitement: *p =* .04). For the disorganization factor, the CON subdivision showed a significantly lower coefficient compared with the DMN (*p =* .03) and AUD (*p =* .04) subdivisions. For the negative factor, the DMN subdivision showed a greater coefficient than either the DAT (*p =* .005) or CON (*p =* .04) subdivisions. For the positive factor, the DMN subdivision showed a greater coefficient than either the DAT (*p =* .05) or CON (*p =* .02) subdivisions, and the DAT subdivision showed a lower coefficient compared with the associative subdivision (*p* = .04) and whole striatum (*p =* .05). It is of note that for no symptom-subdivision relationship did any of the anatomical subdivisions differ significantly from one another.

### Patient–Control Subject Differences in Striatocortical Connectivity and Ki^cer^

When investigating differences between groups in terms of striatal connectivity differences, no significant differences were found between groups in terms of the mean connectivity for any of the defined subdivisions (*p* > .3 for all subdivisions) (Figure S8). There were also no differences between patients and control subjects in terms of subdivision spatial distribution of connectivity (*p* > .3 for all comparisons) (see Supplement).

Differences between patients and control subjects in terms of Ki^cer^ were also examined. No statistically significant differences were observed between patients and control subjects for any subdivision (Figure S9). In a post hoc analysis based on findings in an overlapping cohort that raised dopamine synthesis capacity is present only in responders to antipsychotic treatment (34), we restricted the analysis to those characterized as antipsychotic responder (*n =* 11). In this subgroup, dopamine synthesis capacity was higher compared with control subjects for the DMN (*t*_30_ = 2.78, *p =* .009) and DAT (*t*_30_ = 2.31, *p =* .028) Ki^cer^s (see Table 2).

**Table 2:** Patient–Control Subject Comparisons for Subdivision Ki^cer^ Values

Subdivision (Ki^cer^_∗_10^2^)	Control Subjects (*n* = 21)	Patients (*n* = 29)	Responders (*n* = 11)	*p* Value (Control Subject–Patient) (*df* = 48)	*p* Value (Control Subject–Responder) (*df* = 30)
Whole Striatum	1.29 (0.11)	1.28 (0.10)	1.34 (0.08)	.78	.16
Associative Striatum	1.29 (0.11)	1.29 (0.10)	1.35 (0.088)	.73	.14
Limbic Striatum	1.29 (0.12)	1.26 (0.10)	1.31 (0.08)	.45	.54
Sensorimotor Striatum	1.30 (0.14)	1.30 (0.10)	1.35 (0.10)	.98	.20
AUD	1.22 (0.15)	1.21 (0.18)	1.20 (0.18)	.27	.85
CON	1.33 (0.20)	1.20 (0.14)	1.36 (0.15)	.61	.66
DAT	1.22 (0.18)	1.28 (0.15)	1.34 (0.15)	.24	.028
DMN	1.12 (0.16)	1.17 (0.16)	1.25 (0.09)	.27	.009
SMN	1.28 (0.15)	1.24 (0.15)	1.27 (0.13)	.30	.75

Data are expressed as mean (SD). The *p* values were calculated using an independent samples *t* test.

AUD, auditory network; CON, cingulo-opercular network; DAT, dorsal attention network; DMN, default mode network; SMN, sensorimotor network.

## Discussion

In the current study, we describe a novel method for integrating rs-fMRI and ^18^F-DOPA PET in order to derive measures of dopamine function from connectivity-defined striatal subdivisions. The indices of dopamine function calculated using these connectivity-defined subdivisions demonstrated good reliability and also show significantly greater orthogonality compared with anatomical defined subdivisions. Using this approach, we found a strong positive association between the severity of negative symptoms and dopamine synthesis capacity within regions of the striatum functionally linked to the DMN.

While previous studies have investigated the relationship between striatal dopamine function and symptoms in psychotic disorders, this has predominantly been at the level of the whole striatum, and so has not addressed the question of subdivision-specific relationships (13,34,45). Although more recent studies have examined subdivisions, the typical approach employed precludes investigation of the current hypothesis owing to the high degree of collinearity between anatomically defined subdivisions. We demonstrated significantly greater orthogonality in our connectivity-based approach, allowing, to our knowledge for the first time, subdivision-specific relationships to be investigated. This is illustrated by the fact that several symptom-subdivision relationships were significantly different from each other when examining connectivity-defined subdivisions, but no significant differences were observed when examining anatomically defined subdivisions. The greater orthogonality observed with the connectivity-based approach is a natural consequence of the variance induced by the integration of the rs-fMRI data. We demonstrated, however, that this does not come at the expense of significantly reduced reliability. In the case of an anatomical parcellation, one may expect a greater number of voxels within a subdivision to increase reliability, and this is supported by the fact that the largest subdivision (associative striatum) shows the greatest reliability. This, however, is not the case for the connectivity-based approach. In the connectivity-based approach, for each subdivision the entire striatum is sampled. Although the sum of fractional weights for a given connectivity-defined subdivision might appear to be analogous to the total number of voxels in an anatomical defined subdivision, this is not the case and does not show the same relationship with reliability. For example, if one considers a toy example in which each voxel in a connectivity-based subdivision has the same identical weighting of <<1, here the subdivision will possess a low sum of fractional weights, yet its reliability will be equivalent to the entire striatum using an anatomical approach. Indeed, this is one potential reason why greater orthogonality may occur without costing reliability.

In contrast to our connectivity-based approach, traditional anatomically defined subdivisions do not take into account the likely considerable spatial variability in striatal functional specificity that occurs between participants. A connectivity-based approach may be able to account for some of the wide variety that is apparent in terms of striatal volume, shape, and connectivity (48,49). There are likely, however, both advantages and disadvantages to this approach when one considers that schizophrenia is associated with altered functional corticostriatal connectivity (9), and the fact that both corticostriatal (50) and corticocortical (36) connectivity appear to show a relationship with striatal dopamine function. The optimal approach is therefore likely to depend on the scientific question of interest.

The DMN predominantly mapped onto striatal areas that had been defined as associative based on their connection to cortical regions broadly involved in cognition (30). Dopamine dysfunction within this region showed an association with the severity of negative/cognitive symptoms. Recent work including both preclinical studies (15,51) and computational modeling (52) has illustrated how excessive dopamine signaling within the striatum may underlie negative and cognitive symptoms, via a range of mechanisms including impairments in probabilistic learning and disruptions of corticostriatal communication (9). The fact that antipsychotics are relatively ineffective in the treatment of negative symptoms is consistent with these models, as although dopamine antagonism reduces aberrant signaling, it also reduces adaptive signaling, thereby potentially exacerbating negative symptoms (52). While it can be hard to determine in first-episode cohorts whether negative symptoms are secondary to positive symptoms, the Marder factors we used mitigate this by maximizing the orthogonality of symptom clusters. Previous PET studies have been inconsistent in their findings regarding the relationship between striatal dopamine and negative symptoms. A large proportion of previous DOPA PET studies have not reported the relationship with negative symptoms (45,53, 54, 55, 56, 57, 58), and those that have often involved low sample sizes and only stated that statistical significance was not observed (59, 60, 61, 62). Of those that have reported correlation coefficients, a study by Nozaki *et al.* (63) (*N =* 18) found a statistically nonsignificant positive relationship between striatal dopamine synthesis capacity and negative symptoms similar to current finding, while a study by Hietala *et al.* (64) (*N =* 10) reported a nonsignificant negative relationship. Studies using challenge or depletion paradigms are also inconsistent. One PET study using a depletion paradigm found a relationship between greater negative symptom severity and reduced synaptic dopamine levels in the ventral striatum but did not correct for the multiple subdivisions investigated (25), while another study using the same methods found no significant relationship with synaptic dopamine levels within the whole striatum (65). Differences with the current study may result from marked differences in experimental technique, and the fact that the current study included only first-episode patients while the cohort displaying the negative relationship mostly consisted of chronically ill patients. However, given the exploratory nature of these analyses, we suggest that the current findings warrant further testing in new cohorts.

As reported previously, we did not observe a significant difference between patients and control subjects in terms of striatal Ki^cer^ (35). This may represent a type II error, likely exacerbated by the fact that our cohort included a number individuals who were nonresponsive to antipsychotic treatment, a characteristic associated with normal Ki^cer^ (34,45), and indeed when excluding nonresponders, there were significant group differences in Ki^cer^s for the DMN and DAT subdivisions.

Future work would benefit from more detailed behavioral assessment, and larger sample sizes would help reduce the risk of both type I and type II error. Reliability studies would ideally use test-retest MRI and PET scans from the same individuals, as our approach may have underestimated the reliability of the connectivity-based method. Only 8 individuals contributed to the PET test-retest data, and as such, the generalizability of these findings may be limited; however, if reliability was found to be lower in a larger dataset, there is no reason to assume that this would have greater impact on the connectivity-based method compared with an anatomical parcellation. Obtaining PET and MRI measures simultaneously with combined PET-MRI may improve the signal-to-noise ratio. Further work establishing reliability is required, and it would be of interest to explore alternative methods for parcellating the striatum, not only using resting-state data (28,66) but also mapping anatomical connectivity using diffusion tensor imaging (67), which might show greater stability. It should be noted that a large number of nodes were assigned to the DMN, including several that would potentially have been assigned to the frontoparietal network if a different community detection algorithm had been employed. There is no single optimal method for either node definition or community assignment, yet different approaches are likely to have a marked impact on results, potentially limiting the generalizability of our findings (68). The reliability of the striatal subdivision defined on the basis of DAT connectivity was poor, and alternative methods of defining striatal connectivity on an individual basis may lead to improvements here.

In conclusion, we have demonstrated a novel method for generating individualized striatal parcellations and demonstrated some advantages over existing methods, although further validation in future studies is necessary. We showed that dopamine synthesis capacity was particularly aberrant within regions of the striatum linked to the DMN, and that dopamine dysfunction here was strongly associated with the severity of negative symptoms.
