# Supplementary material for: The Topography of Striatal Dopamine and Symptoms in Psychosis: An Integrative Positron Emission Tomography and Magnetic Resonance Imaging Study
Source: Biol Psychiatry Cogn Neurosci Neuroimaging. 2020 Nov;5(11):1040–51. doi: 10.1016/j.bpsc.2020.04.004 (PMC7645803; doi:10.1016/j.bpsc.2020.04.004)
Supplement: Supplementary Materials [file mmc1.pdf]

The Topography of Striatal Dopamine and Symptoms in Psychosis: An Integrative Positron Emission Tomography and Magnetic Resonance Imaging Study

Supplementary Information

SUPPLEMENTAL METHODS 2

ETHICS 2
PARTICIPANTS 2
18F-DOPA PET DATA ACQUISITION AND ANALYSIS 2
MRI ACQUISITION AND PREPROCESSING 3
CORTICAL NETWORK ASSIGNMENT 4
FIGURE S1 SINGLE SUBJECT KICER MAP 5
FIGURE S2 CORTICAL NETWORK ASSIGNMENT 6
STRIATAL PROBABILISTIC PARCELLATION 7
FIGURE S3 STRIATAL PROBABILISTIC CONNECTIVITY MAPS 7
PET-MRI INTEGRATION 8
RELIABILITY ANALYSIS 8
SPATIAL DISTRIBUTION ANALYSIS 8
FIGURE S4 SPATIAL DISTRIBUTION ANALYSIS 9

SUPPLEMENTAL RESULTS 10

CORTICAL NETWORK ASSIGNMENT 10
TEST-RETEST RELIABILITY OF MARTINEZ AND CONNECTIVITY BASED METHODS 11
TEST-RETEST RELIABILITY OF rMRI ONLY 11
FIGURE S5 RELATIONSHIP BETWEEN DOPAMINE SYNTHESIS CAPACITY AND SYMPTOMS (REMOVAL OF MINIMALLY TREATED PARTICIPANTS) 11
FIGURE S6 RELATIONSHIP BETWEEN DOPAMINE SYNTHESIS CAPACITY AND SYMPTOMS (INCLUSION OF VISUAL NETWORK) 12
FIGURE S7 RELATIONSHIPS BETWEEN DOPAMINE SYNTHESIS CAPACITY AND SYMPTOMS (CORTICAL NODE LEVEL PERMUTATIONS) 12
FIGURE S8 COMPARISON OF PATIENTS AND CONTROL CONNECTIVITY OF STRIATAL SUBDIVISIONS 13
PATIENT-CONTROL DIFFERENCES IN SPATIAL DISTRIBUTION OF SUBDIVISIONS 13
FIGURE S9 COMPARISON OF PATIENTS AND CONTROLS RESTING STATE DEFINED KI^{CER} 14

SUPPLEMENTAL REFERENCES 15

Supplemental Methods

Ethics

This study was approved by the East of England-Cambridge East NHS Research Ethics Committee, and Administration of Radioactive Substances Advisory Committee (ARSAC). All participants provided informed written consent to participate.

Participants

Participants were recruited from clinical services in South and West London. Exclusion criteria for all subjects were: history of significant head trauma, dependence on illicit substances, medical co-morbidity (other than minor illnesses), lifetime use of antipsychotic drugs for longer than two weeks,[1] contra-indications to PET and MRI scanning (such as pregnancy), or prescription of mood stabilizer medication. Participants were classified by antipsychotic exposure as antipsychotic naïve, antipsychotic free (prior oral antipsychotic medication but free of treatment for at least 6 weeks (oral) or 6 months (depot, if relevant)) or minimally treated (taking antipsychotic medication for two weeks or less). Participants were assessed by a consultant psychiatrist using the Positive and Negative Syndrome Scale (PANSS)[2]. Of the two participants that had been minimally treated one was prescribed risperidone 2mg, and the other amisulpride 200mg.

18F-DOPA PET Data Acquisition and Analysis

Participants were not permitted to smoke or consume caffeine for four hours preceding the scan. After acquiring a CT scan for attenuation correction, PET images were acquired using a Siemens Biograph HiRez XVI PET scanner (Siemens Healthcare, Erlangen, Germany) at Imanova Centre for Imaging Sciences.

One hour prior to scanning, participants received 400mg entacapone and 150mg carbidopa, to prevent formation of radiolabelled metabolites and reduce peripheral metabolism. Approximately 160 MBq of ^{18}F-DOPA was administered by bolus intravenous injection. The quantification pipeline was consistent with previous works.[3] Correction for head movement during the scan was performed by denoising the non- attenuation-corrected dynamic images using a level 2, order 64 Battle-Lemarie wavelet filter. Frames were realigned to a single reference frame, acquired 20 minutes post-injection, employing a mutual information algorithm.[4,5] The transformation parameters were then applied to the corresponding attenuated-

corrected dynamic images, creating a movement-corrected dynamic image, which was used in the analysis. Realigned frames were then summated to create an individual motion-corrected reference map for the brain tissue segmentation. The cerebellum was used as a reference region (defined as per Hammers et al[6]), and K_i^{cer} was calculated with the Patlak-Gjedde graphical approach adapted for reference tissue input function.[7] Image processing and quantification was done using in-house code with MATLAB 2012b, and SPM8 (Wellcome Trust Centre for Neuroimaging) was used to automatically normalize a tracer-specific template.[8]

In order to generate the voxelwise Ki maps we implemented a previously established method[9] in which Ki^{cer} parametric images of the brain were constructed from motion-corrected images using a wavelet-based approach.[10] The parametric image for each participant was then normalized into Montreal Neurological Institute standard space (matrix dimension: 91x109x91; voxel size: 2mm isotropic) using the participant's PET summation image and the ^{18}F-DOPA template.

MRI Acquisition and Preprocessing

Participants also received a rfMRI scan on a 3T GE Signa MR scanner. Functional imaging consisted of T2* weighted echo planar image slices. 256 volumes were acquired, consisting of 39 interleaved slices (3.5 mm slice thickness, 3.75 mm x 3.75 mm voxel dimensions in plane) with a repetition time (TR) of 2000 ms, and echo time (TE) 30 ms. A structural image was obtained using a gradient-echo scan (TR=7.0s, TE=2.8s, flip angle=11°, in plane resolution=1mm x 1mm, slice thickness=1.2mm, 196 slices).

Image pre-processing was performed using a standard pipeline implemented in the CONN toolbox (version 17.b)[11] for Statistical Parametric Mapping software (SPM 12 (6906)). A standard preprocessing pipeline was used consisting of slice timing correction, realignment, and normalisation to MNI space. Images were smoothed with a Gaussian kernel of 8mm full-width-half-maximum. The ART toolbox was used to account for motion and artefact detection using anatomical component based correction (aCompCor) of temporal confounds relating to head movement and physiological noise. This method models noise effects at a voxel level based on estimates derived from principal components of noise regions of interest (white matter and CSF, eroded by one voxel to minimise partial volume effects), and then removes these from the BOLD timeseries using linear regression. Six residual head motion parameters and their first order temporal derivatives were also entered as regressors into the first level model.

A confounding effect accounting for magnetisation stabilisation, and its first order derivative was entered. A scrubbing procedure was also implemented in that artifact/outlier volumes (average intensity deviated more than 5 standard deviations from the mean intensity in the session, or composite head movement exceeded 0.9 mm from the previous image) were also regressed out. Preprocessed data were temporally bandpass filtered (0.008-0.09 Hz)

Cortical Network Assignment

Time series were extracted from the 333 nodes of the Gordon atlas.[12] The Gordon parcellation was developed using resting state boundary maps observed in a sample of 120 healthy young adults, it shows superior within parcel homogeneity in comparison to other parcellations, making it an appropriate choice for the analysis of resting state data. For each participant, a 333x333 connectivity matrix (also termed a *graph*) was constructed where each edge between two nodes represents the z-transformed Pearson correlation coefficient of the time series of these nodes.

A group averaged connectivity matrix was then constructed. To do this we first rescaled each participant's graph, by subtracting from each edge that participant's average edge strength, and dividing by the standard deviation of the strength of all that graph's edges. As a result, all participants then have the same average graph connectivity strength, and so individuals with greater average connectivity values do not have undue influence on the overall group level graph.

We then ran the Louvain community detection algorithm on the whole brain group level graph,[13] in order to assign individual nodes to networks based on the connectivity patterns present in the current dataset. Due to the non-deterministic nature of the Louvain algorithm, a previously described consensus clustering approach was employed.[14,15] Edges between nodes closer than 10mm were discarded (euclidean minimum distance between two closest points of the two nodes), and the strongest 5% of edges were retained and binarised. The gamma parameter was set to 1.4. Networks defined by this approach were then labelled according to whichever of the apriori defined networks they showed the greatest overlap with. This identified the default mode, sensorimotor, cinguloopercular, dorsal attention, auditory and visual networks (see Figure S1). The visual network was excluded in subsequent analysis given its relative lack of direct connections with the striatum.[17]

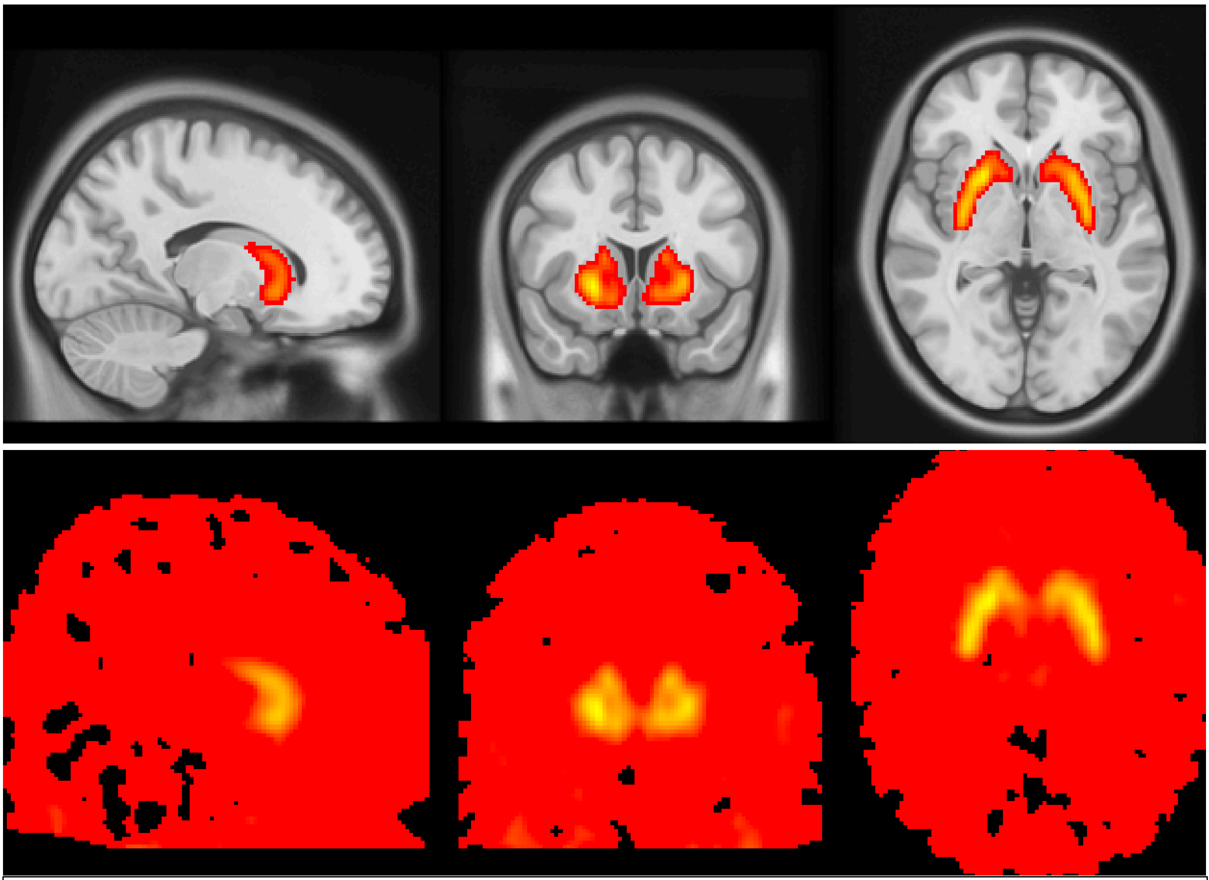

Figure S1 Single Subject Kicer Map
The top image is thresholded to remove values <0.009 and overlaid on a MNI T1 template. The bottom image is the same map without thresholding.

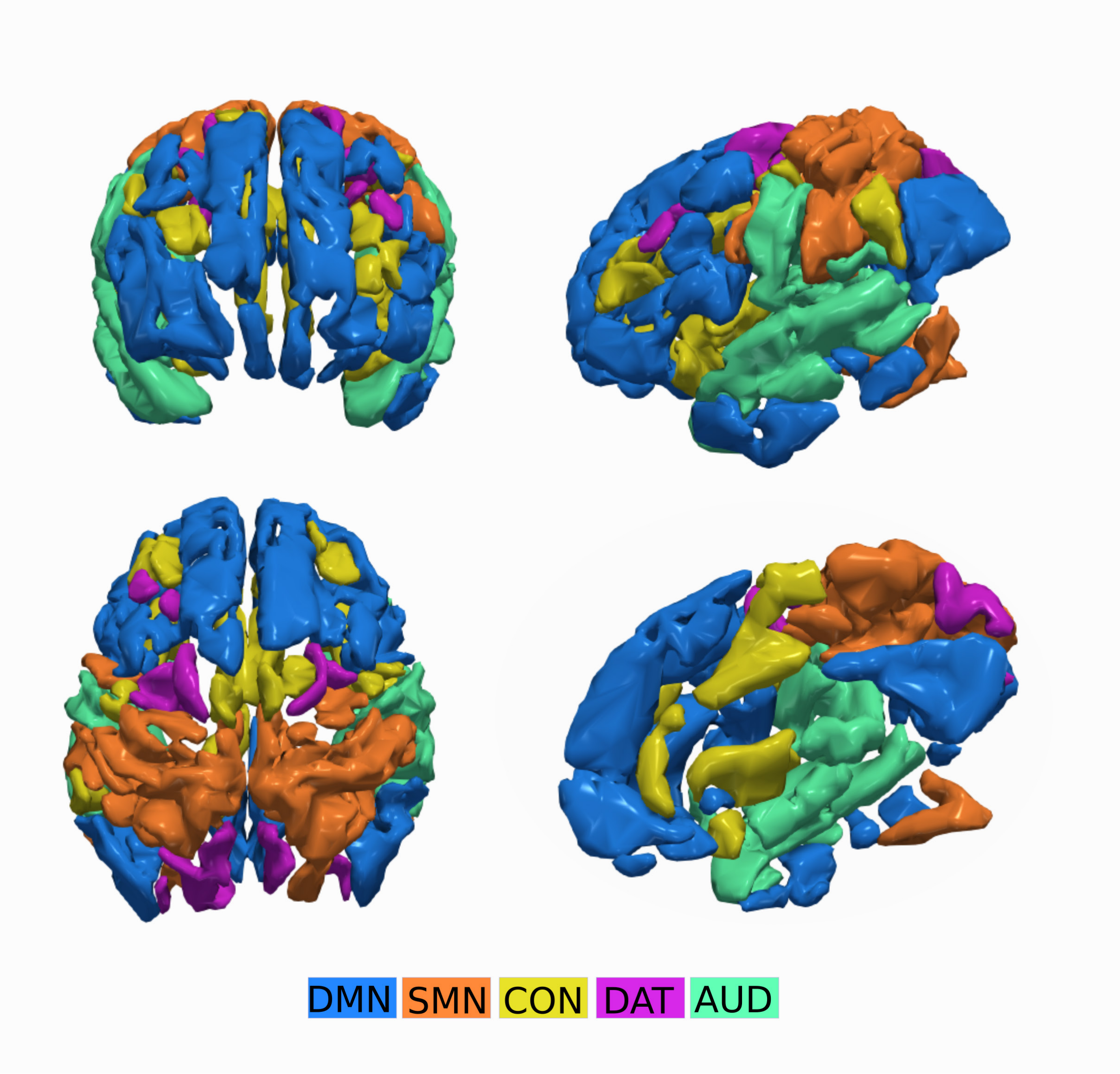

Figure S2 Cortical Network Assignment
AUD- Auditory, CON – Cinguloopercular, DAT – Dorsal attention, DMN – Default mode, SMN – Sensorimotor.

Striatal Probabilistic Parcellation

For each striatal voxel the z-transformed correlation coefficient between the voxel and the 333 Gordon nodes was calculated. Then for each of the networks defined in the *Network Assignment* step above the mean connectivity of that network's nodes to the voxel was calculated, with negative values being set at 0. When this had been performed for each network these values were then scaled so that at each voxel the sum of the five connectivity values (one for each network) equalled 1 (see Figure S2).

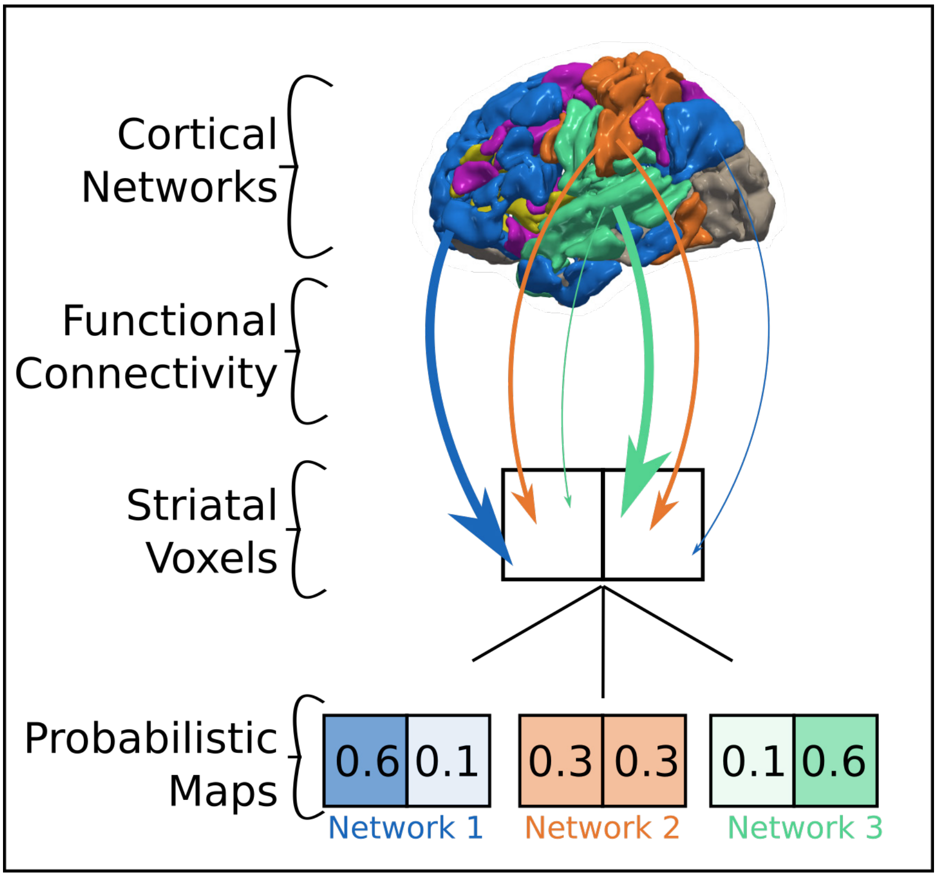

Figure S3 Striatal Probabilistic Connectivity Maps

Each striatal voxel is assigned a value for each of the cortical networks, based on the mean connectivity of the voxel to each node in the network. The total connectivity score for each voxel sums to 1. In the example above the left hand voxel shows is weighted most strongly for network 1, and least for network 3.

PET-MRI Integration

At the individual participant level for each network, for each striatal voxel we multiplied the Ki^{cer} value at that voxel by the weight assigned to that network at that voxel. We then averaged across voxels to generate for each network a network specific Ki^{cer}.

Reliability Analysis

Test-retest resting state MRI data was obtained from the Human Connectome project.[18] We removed 40% of the volumes from these scans so that the scan length matched that of our own data. For details of the test-retest PET data please see the previously published report.[19] Striatal parcels were defined in the same manner as for the main dataset, normalisation of these MNI parcels into individual patient space was then performed as described in the original test-retest paper[19].

Spatial Distribution Analysis

In order to determine if patients and controls differed in terms of the spatial distribution of the connectivity based subdivisions we performed an analysis as illustrated in Figure S3.

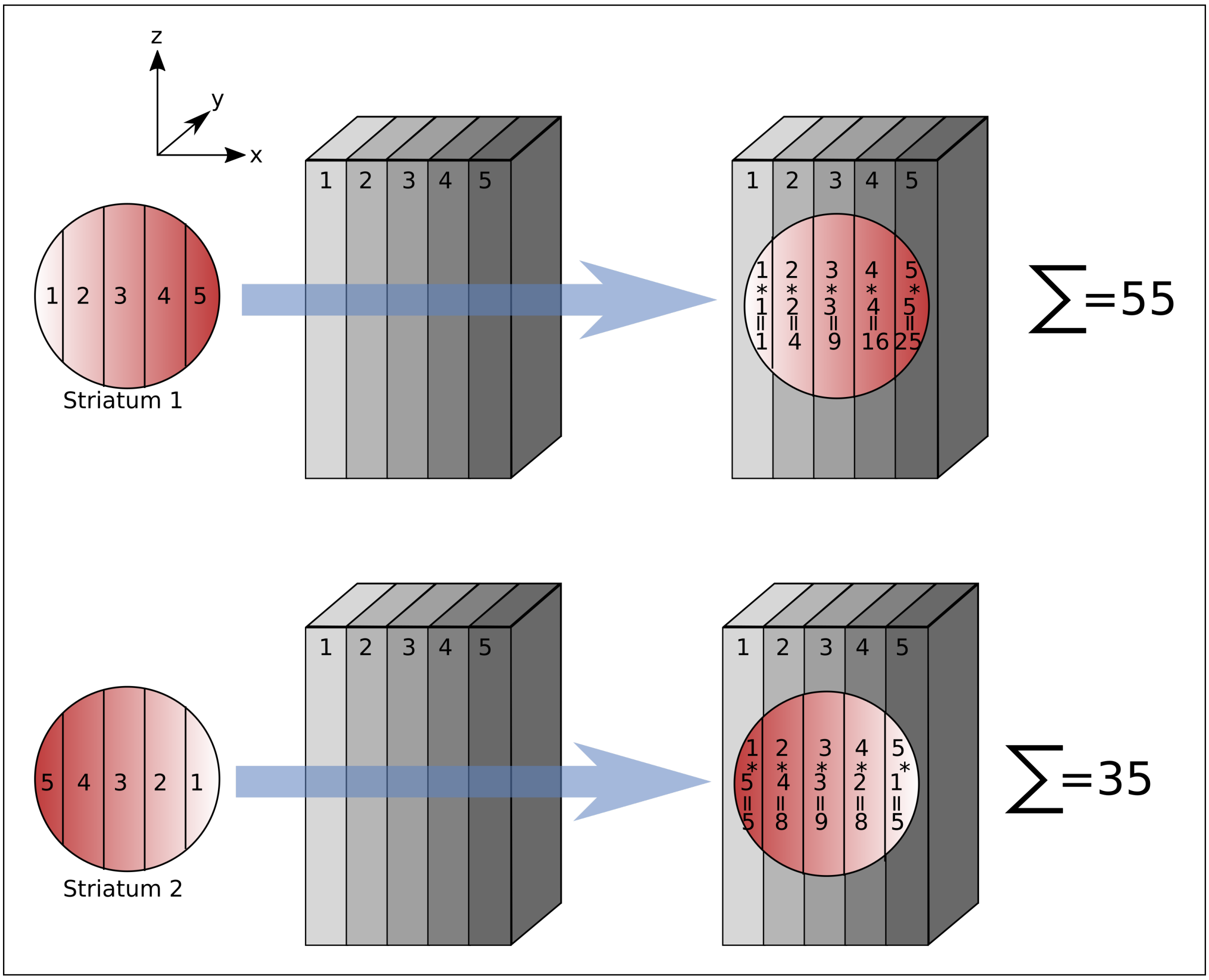

Figure S4 Spatial Distribution Analysis

Example of quantifying the extent to which a striatal map is weighted along the x-axis. The voxel intensities of the striatum are multiplied (entrywise product) by a matrix linearly increasing along the x-axis, and the result is summed. Striatum 1 is weighted towards the right, and so scores higher than striatum 2 that is weighted towards the left.

Supplemental Results

Cortical Network Assignment

Numbers refer to the node labels provided by Gordon et al.[12]

Default = [1, 4, 6, 9, 23, 24, 25, 44, 60, 61, 74, 75, 78, 79, 85, 86, 94, 96, 107, 108, 109, 112, 113, 114, 115, 116, 119, 120, 126, 128, 129,133,145,146,148, 150,151,156,157, 158,281,300, 301,124,162,165,167,168,170,182,186,200,219,220,236,237,240,241, 247, 250, 259,260,261,267,272,273,275,276,277,278,279,283,289,291,302,315,316,319,320, 321,322,323,324,325,326,327,328]

Sensorimotor = [37, 38, 45, 47, 50, 51, 52, 54, 55, 56, 57, 58, 59, 88,100,104,105,106, 163,203,205,209,210,211,213,215,217,218,251,252,262,265,266,270, 2, 30, 31, 32, 33, 35, 36, 46,180,190,191,193,194,195,201,202,204,206,214,216]

Cingulopercular = [7, 12, 21, 22, 26, 27, 28, 29, 34, 39, 40, 63, 76, 80, 81, 82, 83, 84,110,111,117,147,149,152,153,181,183,184,185,187,188,192,196,198,238,243,245,246,24 8,249,271,274,317,318]

Dorsal Attention = [41, 42, 43, 48, 49, 87, 91, 92, 93, 95,154,155,189,199,207,208,253,257]

Auditory = [64, 3, 10, 11, 53, 62, 65, 66, 67, 68, 69, 70, 71, 72, 73, 77,101,102,103,127, 159,160,161,164,171,172,197,212,221,222,223,224,225,226, 227,228,229,230,231,232,233,234,235,239,242,244,268,269,290,292,329,330,331,332,333]

Test-retest Reliability of Martinez and Connectivity Based Methods

Parcellation Method	Network	ICC mean (SD)
Resting State Parcellation	DMN	0.75 (0.10)
	SMN	0.74 (0.11)
	CON	0.74 (0.06)
	DAT	0.73 (0.12)
	AUD	0.78 (0.05)
Martinez Parcellation	Limbic	0.66
	Associative	0.80
	Sensorimotor	0.65

Test-retest Reliability of rMRI Only

Parcellation Method	Network	ICC
Resting State Parcellation	DMN	0.54
	SMN	0.65
	CON	0.48
	DAT	0.32
	AUD	0.64

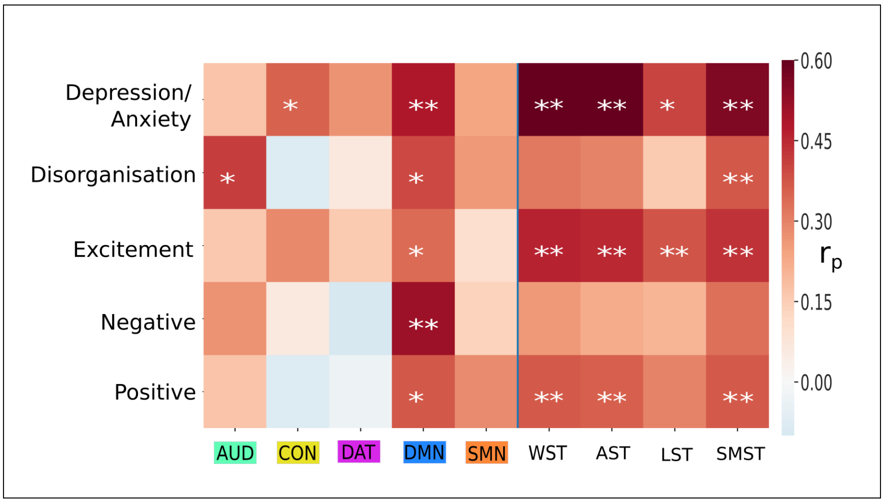

Figure S5 Relationship Between Dopamine Synthesis Capacity and Symptoms (Removal of Minimally Treated Participants)

Relationships between symptoms and subdivision Ki values remained similar following the removal of the two minimally treated participants (*=p<0.05, **=p<0.05 FDR corrected)

Connectivity defined subdivisions: AUD- Auditory , CON – Cingulopercular, DAT – Dorsal attention, DMN – Default mode, SMN – Sensorimotor.
Anatomcially defined subdivisions: WST – Whole striatum, AST – Associative Striatum, LST – Limbic Striatum, SMST – Sensorimotor striatum

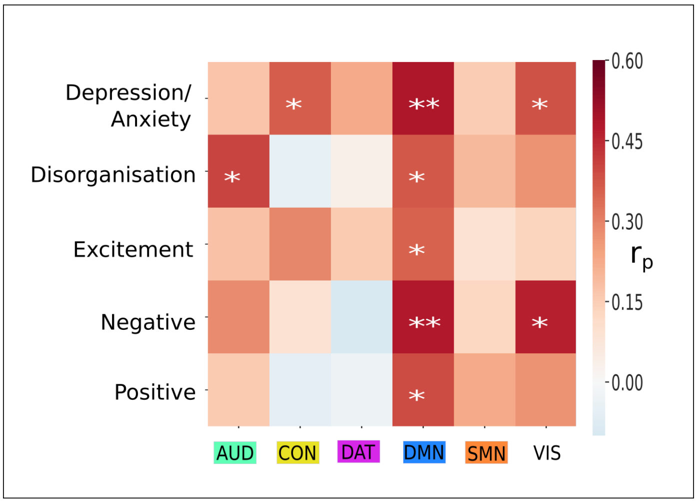

Figure S6 Relationship Between Dopamine Synthesis Capacity and Symptoms (Inclusion of Visual Network)
(*=p<0.05, **=p<0.05 FDR corrected)

Connectivity defined subdivisions: AUD- Auditory, CON – Cingulopercular, DAT – Dorsal attention, DMN – Default mode, SMN – Sensorimotor. VIS - Visual

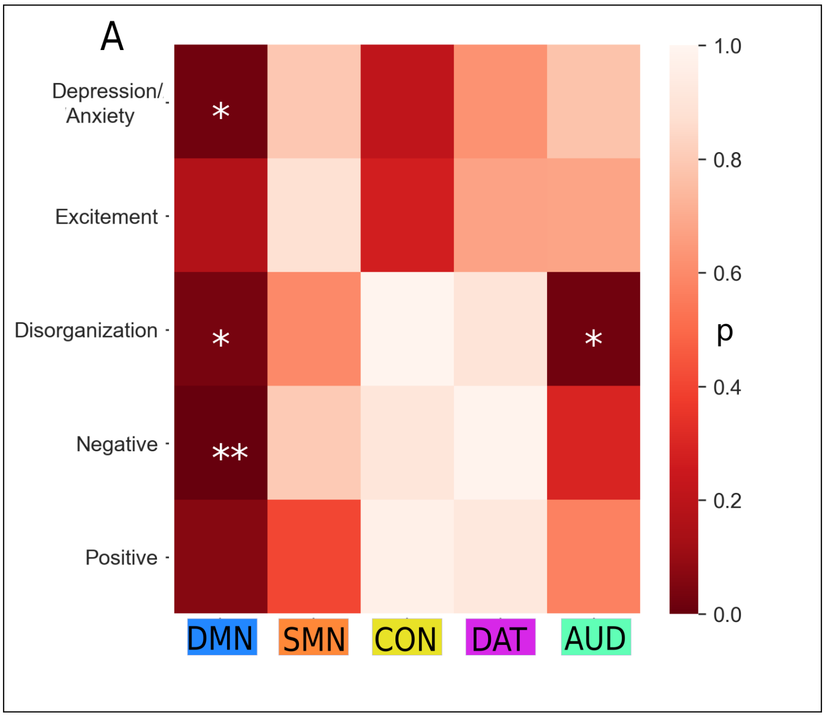

Figure S7 Relationships Between Dopamine Synthesis Capacity and Symptoms (Cortical Node Level Permutations)
Associations between PANSS Marder factors and striatal Ki^{cer} across different connectivity defined striatal regions. Heatmap displays p values calculated using cortical node permutation testing. (*=p<0.05, **=p<0.05 FDR corrected)

AUD- Auditory, CON – Cingulopercular, DAT – Dorsal attention, DMN – Default mode, SMN – Sensorimotor.

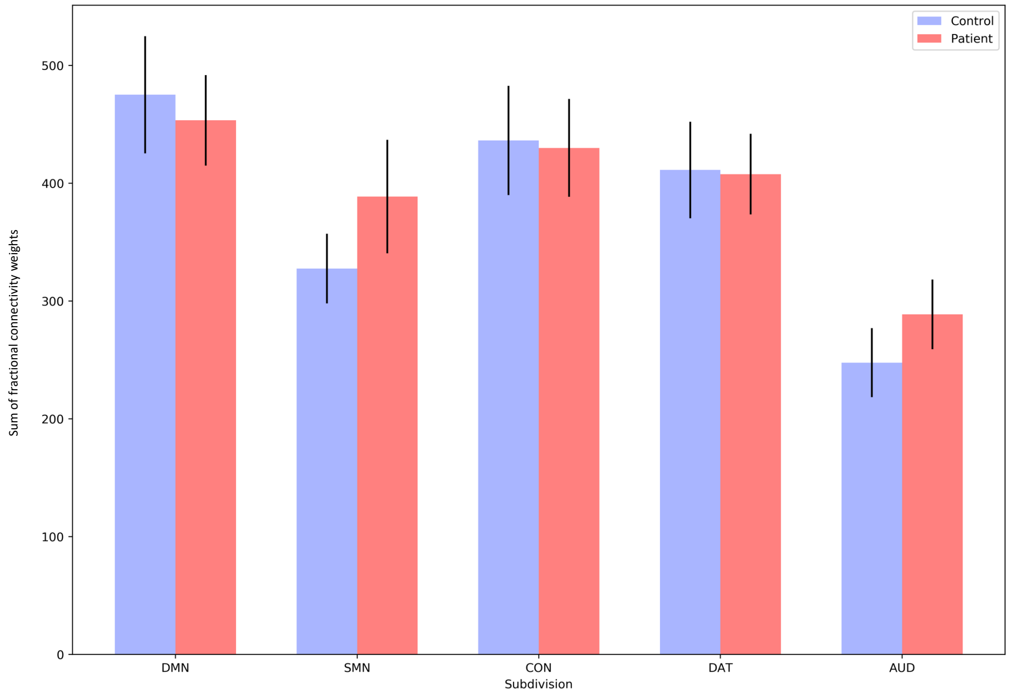

Figure S8 Comparison of Patients and Control Connectivity of Striatal Subdivisions

Average connectivity values within resting state defined striatal subdivisions for patients and controls. There were no significant differences between the groups for any of the subdivisions. Error bars = +/- 1SE

AUD- Auditory, CON – Cingulopercular, DAT – Dorsal attention, DMN – Default mode, SMN – Sensorimotor.

Patient-Control Differences in Spatial Distribution of Subdivisions

Dimension	DMN	SMN	CON	DAT	AUD
X	t=0.21<br>p=0.82	t=0.04<br>p=0.97	t=-0.40<br>p=0.69	t=0.23<br>p=0.82	t=0.91<br>p=0.42
Y	t=0.49<br>p=0.62	t=-0.19<br>p=0.85	t=-0.21<br>p= 0.84	t=0.04<br>p=0.97	t=0.80<br>p=0.43
Z	t=0.11<br>p=0.91	t=0.22<br>p=0.82	t=-0.22<br>P=0.83	t=0.03<br>p=0.97	t=0.90<br>p=0.37

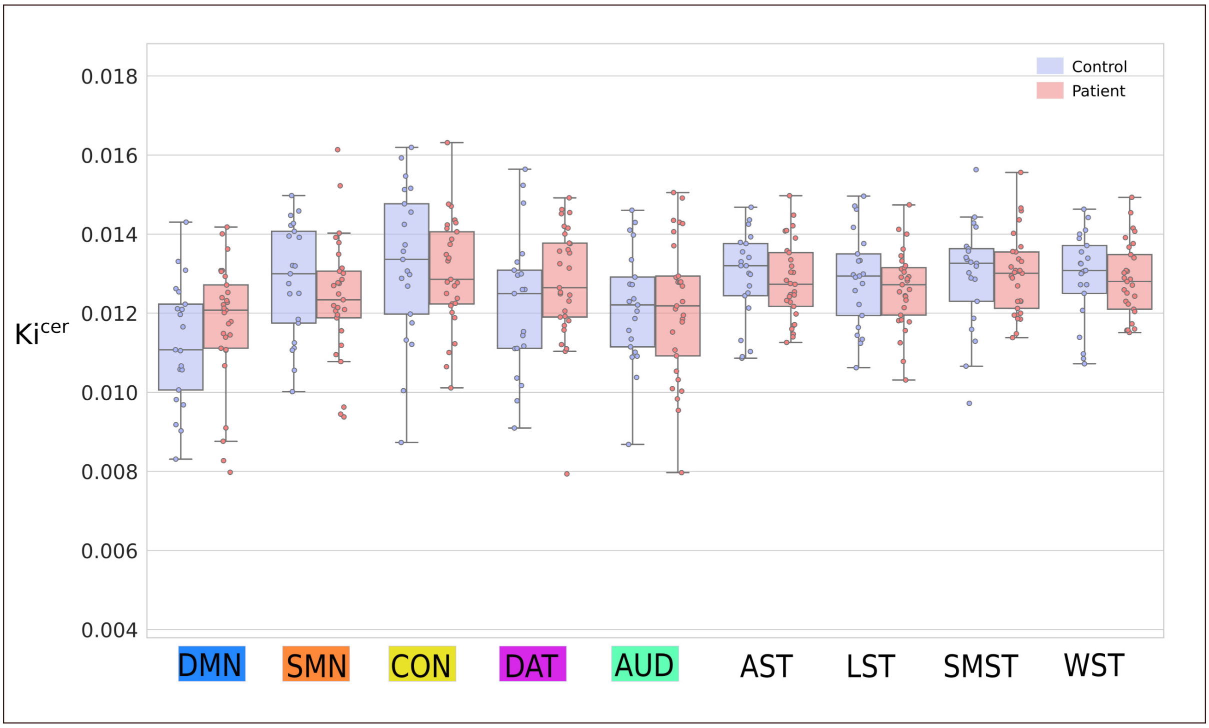

Figure S9 Comparison of Patients and Controls Resting State Defined Ki^{cer}

Average dopamine synthesis capacity within resting state defined striatal subdivisions for patients and controls. There were no significant differences between the groups for any of the subdivisions. Error bars = +/- 1SE

AUD- Auditory, CON – Cingulopercular, DAT – Dorsal attention, DMN – Default mode, SMN – Sensorimotor.
WST – Whole striatum, AST – Associative Striatum, LST – Limbic Striatum, SMST – Sensorimotor striatum

Supplemental References

1 Leucht S, Winter-van Rossum I, Heres S, Arango C, Fleischhacker WW, Glenthøj B *et al.* The optimization of treatment and management of schizophrenia in Europe (OPTiMiSE) trial: rationale for its methodology and a review of the effectiveness of switching antipsychotics. *Schizophr Bull* 2015; 41: 549–558.

2 Kay SR, Flszbein A, Opfer LA. The positive and negative syndrome scale (PANSS) for schizophrenia. *Schizophr Bull* 1987; 13: 261.

3 Jauhar S, Veronese M, Rogdaki M, Bloomfield M, Natesan S, Turkheimer F *et al.* Regulation of dopaminergic function: an [18F]-DOPA PET apomorphine challenge study in humans. *Transl Psychiatry* 2017; 7: e1027.

4 Turkheimer FE, Brett M, Visvikis D, Cunningham VJ. Multiresolution analysis of emission tomography images in the wavelet domain. *J Cereb Blood Flow Metab Off J Int Soc Cereb Blood Flow Metab* 1999; 19: 1189–1208.

5 Studholme C, Hill DL, Hawkes DJ. Automated 3-D registration of MR and CT images of the head. *Med Image Anal* 1996; 1: 163–175.

6 Hammers A, Allom R, Koepp MJ, Free SL, Myers R, Lemieux L *et al.* Three-dimensional maximum probability atlas of the human brain, with particular reference to the temporal lobe. *Hum Brain Mapp* 2003; 19: 224–247.

7 Patlak CS, Blasberg RG. Graphical Evaluation of Blood-to-Brain Transfer Constants from Multiple-Time Uptake Data. Generalizations. *J Cereb Blood Flow Metab* 1985; 5: 584–590.

8 Mcgowan S, Lawrence AD, Sales T. Presynaptic Dopaminergic Dysfunction in Schizophrenia. *Arch Gen Psychiatry* 2004; 61: 134–142.

9 Howes O, Bose S, Turkheimer FE, Valli I, Egerton A, Valmaggia L *et al.* Dopamine synthesis capacity before onset of psychosis: a prospective -DOPA PET imaging study. *Am J Psychiatry* 2011; 168: 1311–1317.

10 Turkheimer FE, Aston JAD, Asselin M-C, Hinz R. Multi-resolution Bayesian regression in PET dynamic studies using wavelets. *Neuroimage* 2006; 32: 111–121.

11 Whitfield-Gabrieli S, Nieto-Castanon A. Conn: A Functional Connectivity Toolbox for Correlated and Anticorrelated Brain Networks. *Brain Connect* 2012; 2: 125–141.

12 Gordon EM, Laumann TO, Adeyemo B, Huckins JF, Kelley WM, Petersen SE. Generation and Evaluation of a Cortical Area Parcellation from Resting-State Correlations. *Cereb Cortex* 2016; 26: 288–303.

13 Blondel VD, Guillaume JL, Lambiotte R, Lefebvre E. Fast unfolding of communities in

large networks. *J Stat Mech Theory Exp* 2008; 2008: 1–12.

14 Lancichinetti A, Fortunato S. Consensus clustering in complex networks. *Sci Rep* 2012; 2. doi:10.1038/srep00336.

15 McCutcheon R, Nour MM, Dahoun T, Jauhar S, Pepper F, Expert P *et al.* Mesolimbic Dopamine Function is Related to Salience Network Connectivity: An Integrative PET and MR Study. *Biol Psychiatry* 2018; : 1–11.

16 Dice LR. Measures of the Amount of Ecologic Association Between Species. *Ecology* 1945; 26: 297–302.

17 Parent A, Hazrati L. Functional anatomy of the basal ganglia. I. THe cortico-basal ganglia-thalamo-cortical loop. *Brain Res Rev* 1995; 20: 91–127.

18 Van Essen DC, Smith SM, Barch DM, Behrens TEJ, Yacoub E, Ugurbil K. The WU-Minn Human Connectome Project: An overview. *Neuroimage* 2013; 80: 62–79.

19 Egerton A, Demjaha A, McGuire P, Mehta MA, Howes OD. The test-retest reliability of 18F-DOPA PET in assessing striatal and extrastriatal presynaptic dopaminergic function. *Neuroimage* 2010; 50: 524–531.
